# Supplementary material for: Multi-level chirality in liquid crystals formed by achiral molecules
Source: Nat Commun. 2019 Apr 23;10:1922. doi: 10.1038/s41467-019-09862-y (PMC6478950; doi:10.1038/s41467-019-09862-y)
Supplement: Supplementary file 1 — Supplementary Information [file 41467_2019_9862_MOESM1_ESM.pdf]

Supplementary information for:

Multi-level chirality in liquid crystals formed by achiral molecules

Salamończyk et al.

Supplementary Figures

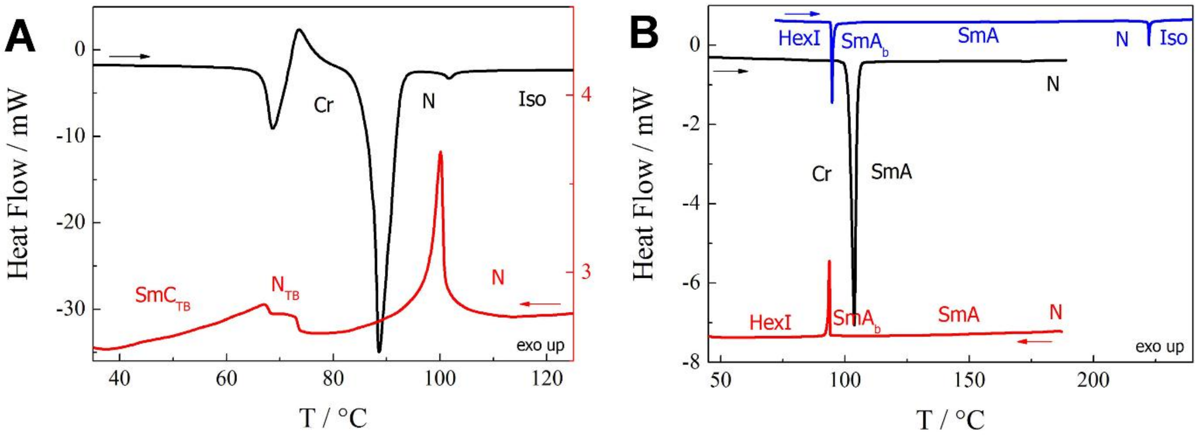

Supplementary Figure 1. DSC thermograms for heating and cooling scans for compounds (A) *D1* and (B) *D2*. Blue line in B shows second heating run, which was started from supercooled $\boldsymbol{HexI}$ phase in order to record monotropic $\boldsymbol{HexI} - \boldsymbol{SmA}_{\mathbf{b}}$ phase transition on heating.

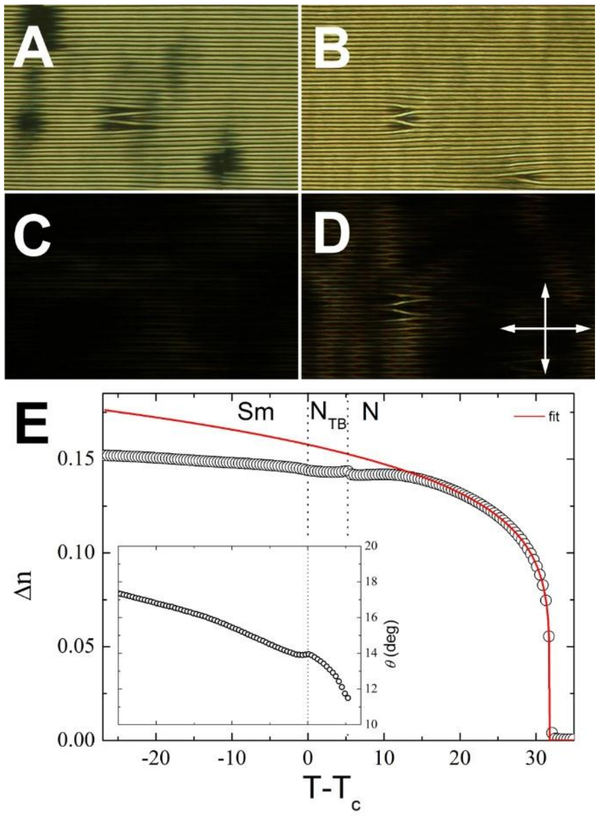

Supplementary Figure 2. Optical stripe texture in (**A**) the twist-bend nematic, N_{TB}, phase and (**B**) smectic phase observed for compound *D1* in a 3 μm thick planar cell. A nearly uniform texture in the (**C**) N_{TB} phase and (**D**) smectic phase grown in sample kept for several minutes at a constant temperature. The polarizer directions are denoted by arrows. (**E**) The birefringence (Δn) of a uniform texture vs. temperature (T) measured on heating with red light (690 nm). The red line represents the fitting to the critical behavior $\Delta n = \Delta n_0\ [(T - T_{Iso-N})/T_{Iso-N}]^{\beta}$ ($\Delta n_0\ =\ 0.24, \beta = 0.18, T_{Iso-N} = 371.65$ K); the decrease of birefringence from the extrapolated Δn value is due to the tilting of the molecules and formation of the heliconical structure. The inset: the conical tilt angle (θ) vs. temperature, calculated from the changes in birefringence.

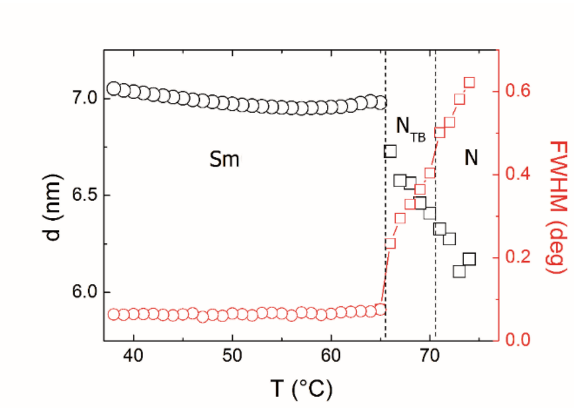

Supplementary Figure 3. Layer thickness ($\boldsymbol{d}$) vs. temperature ($\boldsymbol{T}$) (black) and the width of the signal ($\boldsymbol{FWHM}$) vs. temperature (red) for material *D1*.

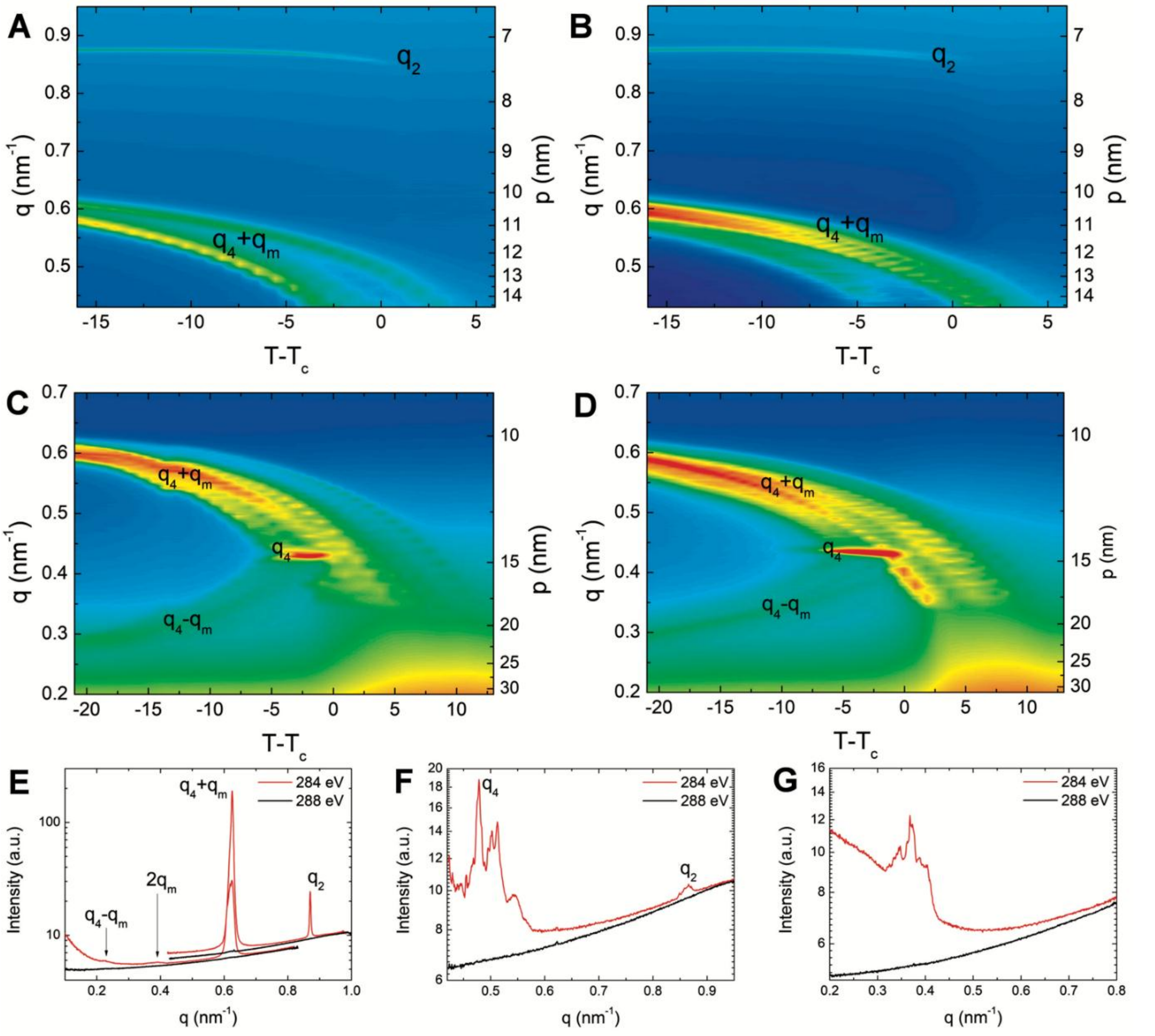

Supplementary Figure 4. The consecutive RSoXS scans for material *D1* obtained at (**A, C**) heating and (**B, D**) cooling covering different scattering vector magnitude (q) ranges, p is the corresponding modulation pitch. The intensity vs. q registered at the resonant energy 284 eV and out of resonance at 288 eV in the smectic phase (**E**) 20 K and (**F**) 3 K below the N_{TB} – smectic phase transition, and in (**G**) the N_{TB} phase 5 K above the N_{TB} – smectic phase transition. The scattering vectors due to the 4-layer and bilayer periodicities are q_4 and q_2, respectively, q_{m} is the modulation wave vector.

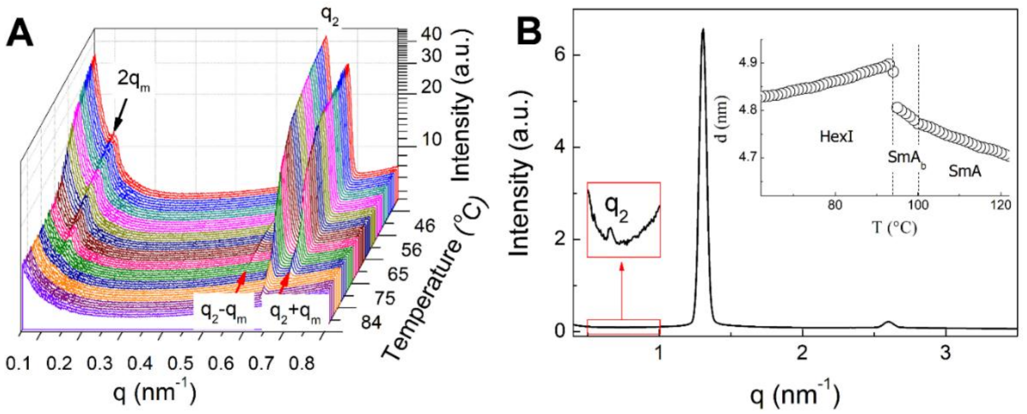

Supplementary Figure 5. (**A**) Intensity vs. scattering wave vector magnitude (q) and temperature (T) for material *D2* in the hexatic phase ($HexI$) obtained from the RSoXS measurements. The q_2 peak corresponding to the bilayer periodicity, a half-pitch modulation peak at $q = 2q_{\mathrm{m}}$ and two peaks at $q = q_2 \pm q_{\mathrm{m}}$ are observed in the $HexI$ phase. (**B**) Intensity vs. q for material *D2* in the hexatic phase obtained from the nonresonant x-ray scattering; the inset: the layer thickness (d) evolution with temperature.

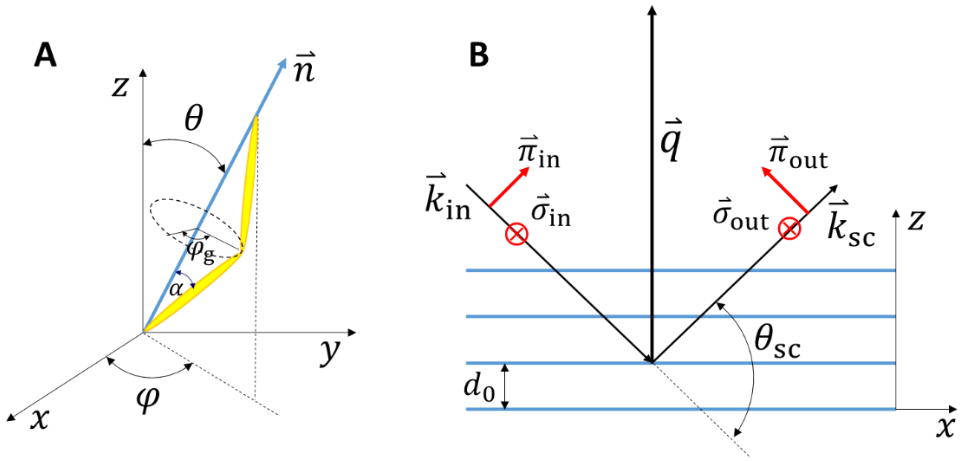

Supplementary Figure 6. (**A**) The orientation of the long molecular axis (defined by the director $\vec{n}$) in the laboratory coordinate system is defined by the tilt angle θ and the azimuthal angle φ. The apex angle of the molecule is $\pi - 2\alpha$ and the direction of the molecular tip with respect to the tilt plane, defined by the director $\vec{n}$ and the smectic layer normal (direction z), is defined by the general tilt angle φ_{g}, which is zero, if the molecular tilt is in the direction perpendicular to the tilt plane in the direction of $\vec{n} \times \hat{z}$. (**B**) The scattering geometry; $\vec{k}_{\mathrm{in}}$ and $\vec{k}_{\mathrm{out}}$ are the wave vectors of the incident and scattered wave, respectively, $\vec{q}$ is the scattering vector; $\vec{\sigma}_{\mathrm{in,out}}$ and $\vec{\pi}_{\mathrm{in,out}}$ are polarizations of the incident and scattered light, θ_{sc} is the scattering angle and d_0 is the smectic layer thickness.

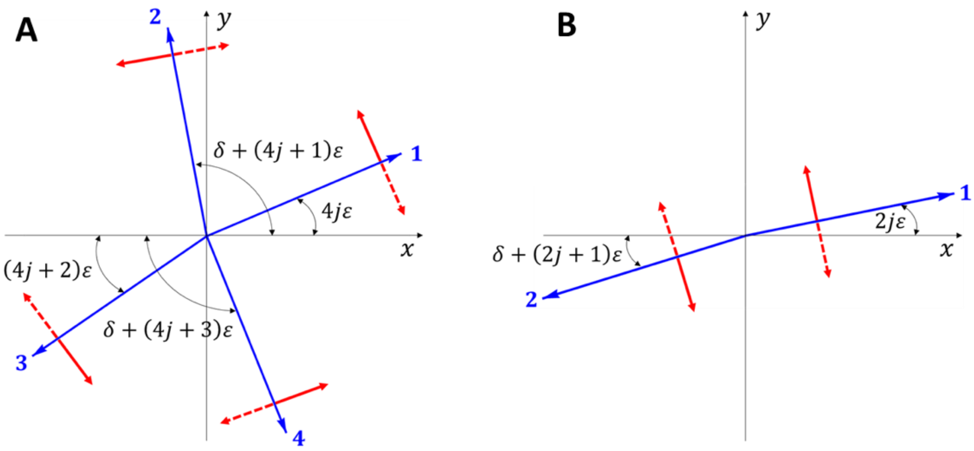

Supplementary Figure 7. (**A**) In the four layer structure the molecules in the successive layers differ in the direction of the long molecular axis direction. The figure shows the projection of the director $\vec{n}$ on the smectic plane (xy-plane) in the j-th stack of four successive layers. The structure is ferri-like: the angle between the director projections in layers 1 and 2 and layers 3 and 4 is δ, and the angle between the layers 1 and 3 and 2 and 4 is π. To this "basic" ferri-like structure an additional rotation by an angle ε is superimposed. (**B**) The bilayer structure. To an almost anticlinic structure of the director in the neighboring layers the additional rotation by angle ε is superimposed. Both figures: Thick red (solid an dashed) arrows denote the direction of the molecular tip when the tilt is perpendicular to the tilt plane (φ_g equals 0 or π).

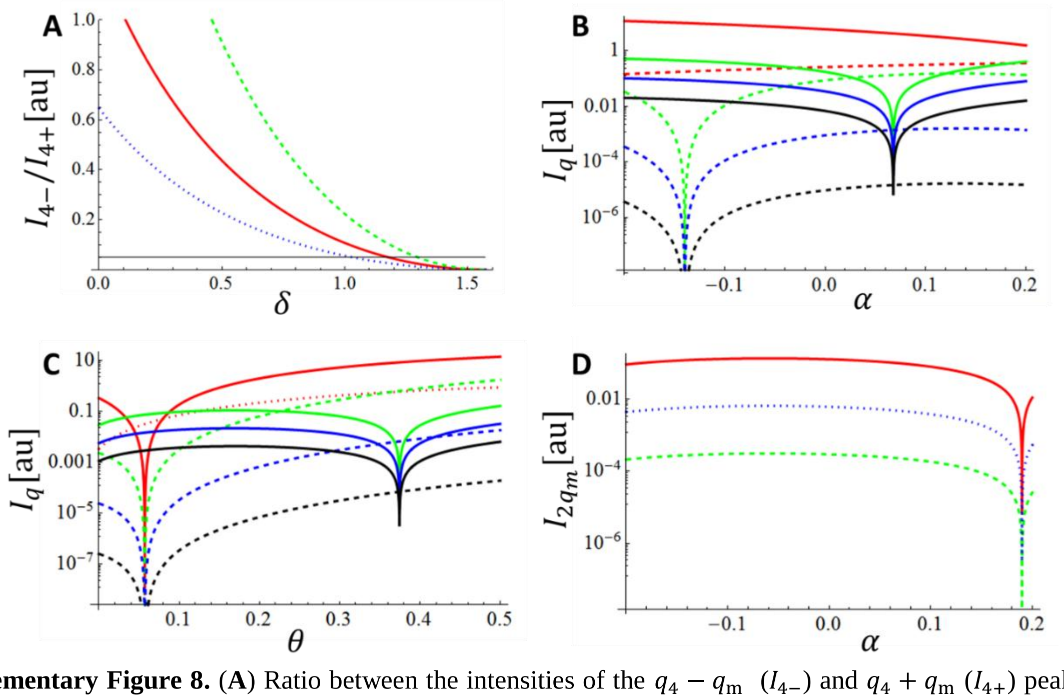

Supplementary Figure 8. (**A**) Ratio between the intensities of the $q_4 - q_{\mathrm{m}}$ (I_{4-}) and $q_4 + q_{\mathrm{m}}$ (I_{4+}) peaks as a function of δ at $\theta = 0.25$ and $\varepsilon = 0.5$ for $\alpha = 0$ (red solid line), $\alpha = -0.1$ (blue dotted line) and $\alpha = 0.1$ (green dashed line). (**B**) The intensities (I_q) of the scattered light as a function of α at $\theta = 0.25$, $\delta = 1.2$ and $\varepsilon = 0.5$ for the peaks at $q = q_4 + q_{\mathrm{m}}$ (red solid line: $\pi\sigma$ or $\sigma\pi$-polarizations), $q_4 - q_{\mathrm{m}}$ (red dashed line: $\pi\sigma$ or $\sigma\pi$-polarizations), $q_2 + 2q_{\mathrm{m}}$ (solid green line: $\sigma\sigma$-polarizations; blue solid line: $\pi\sigma$ or $\sigma\pi$-polarizations; black solid line: $\pi\pi$-polarization) and $q_2 - 2q_{\mathrm{m}}$ (dashed green line: $\sigma\sigma$-polarizations; blue dashed line: $\pi\sigma$ or $\sigma\pi$-polarizations; black dashed line: $\pi\pi$-polarizations). (**C**) The intensities of the scattered light as a function of θ at $\alpha = 0.1$, $\delta = 1.2$ and $\varepsilon = 0.5$ for the peaks at $q_4 + q_{\mathrm{m}}$ (red solid line: $\pi\sigma$ or $\sigma\pi$-polarizations), $q_4 - q_{\mathrm{m}}$ (red dashed line: $\pi\sigma$ or $\sigma\pi$-polarizations), $q_2 + 2q_{\mathrm{m}}$ (solid green line: $\sigma\sigma$-polarizations; blue solid line: $\pi\sigma$ or $\sigma\pi$-polarizations; black solid line: $\pi\pi$-polarization) and $q_2 - 2q_{\mathrm{m}}$ (dashed green line: $\sigma\sigma$-polarizations; blue dashed line: $\pi\sigma$ or $\sigma\pi$-polarizations; black dashed line: $\pi\pi$-polarizations). (**D**) The intensities ($I_{2q_{\mathrm{m}}}$) of the scattered light as a function of α at $\theta = 0.25$, $\delta = 1.2$ and $\varepsilon = 0.5$ for the peaks at $2q_{\mathrm{m}}$; red solid line: $\sigma\sigma$-polarizations; blue dotted line: $\pi\sigma$ or $\sigma\pi$-polarizations; green dashed line: $\pi\pi$-polarizations.

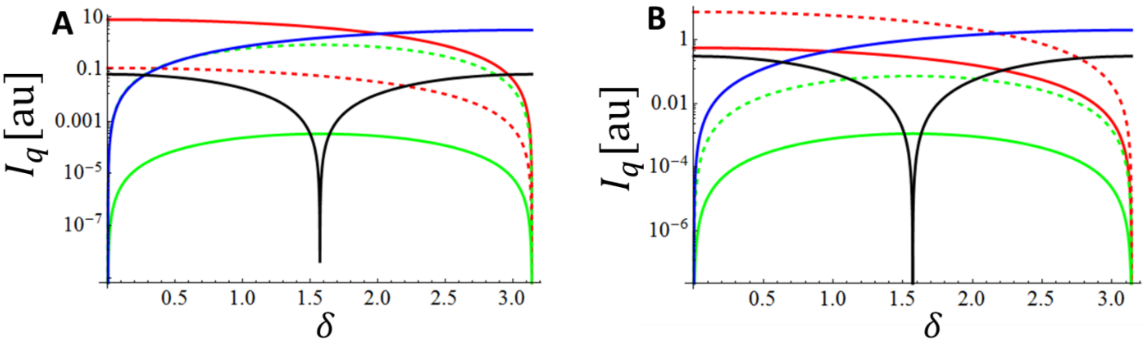

Supplementary Figure 9. The intensities (I_q) of the scattered light as a function of δ at $\theta = 0.4$, $\varepsilon = 0.3$ and (**A**) $\alpha = -\pi/6$ and (**B**) $\alpha = \pi/6$ for the peaks with the scattering wave vector equal to $q_2 + q_\mathrm{m}$ (solid red line), $q_2 - q_\mathrm{m}$ (dashed red line), $q_2 + 2q_\mathrm{m}$ (solid green line), $q_2 - 2q_\mathrm{m}$ (dashed green line), q_m (solid blue line) and $2q_\mathrm{m}$ (solid black line). The $q_2 \pm q_\mathrm{m}$ and q_m peaks are σ-polarized for the π-polarized incident light and vice versa. The intensities of the $q_2 \pm 2q_\mathrm{m}$ and $2q_\mathrm{m}$ peaks are the highest for the σ-polarized incident and scattered light and only these intensities are shown. For other combinations of polarizations the scattered intensity is by at least one order of magnitude lower.

Supplementary Discussion

In modelling the response in the resonant x-ray scattering (*1*, *2*) which provides the information on the orientational structure of molecules, we model a bent-core molecule as having two resonant dipoles with a uniaxial polarizability. The tensor form factor $\underline{F_{\mathrm{ei}}}$ in the eigensystem of the dipole, where the local z-axis is along the direction in which polarizability is largest, is proportional to the anisotropic part of the polarizability tensor (*3*):

$$\underline{F_{\mathrm{ei}}} = f_0 \begin{pmatrix} 1 & 0 & 0 \\ 0 & 1 & 0 \\ 0 & 0 & -2 \end{pmatrix} ,$$

where f_0 is a parameter that depends on the scattering strength. When measuring at the carbon K-edge, several atoms in the molecule respond, and f_0 is expected to be much larger that in case when there is only one resonant atom, like, for example, Sulphur, built into the molecule. The tensor form factor in the laboratory system is obtained by a set of the following rotations of $\underline{F_{ei}}$ (for angle definitions see Supplementary Figure 6a):

- rotation by angle α (lower arm of the molecule) or $-\alpha$ (upper arm of the molecule) around the local axis y;
- rotation by angle φ_{g} around the z-axis;
- rotation by angle θ around the x-axis;
- rotation by angle φ around the z-axis.

The tensor form factor in the laboratory system is thus:

$$\underline{F} = \underline{R_\varphi}\,\underline{R_\theta}\,\underline{R_{\varphi_{\mathrm{g}}}}\,\underline{R_\alpha}\,\underline{F_{ei}}\,\underline{R_\alpha^T}\,\underline{R_{\varphi_{\mathrm{g}}}^T}\,\underline{R_\theta^T}\,\underline{R_\varphi^T} \quad (1)$$

where $\underline{R_\theta}$, $\underline{R_\varphi}$, $\underline{R_{\varphi_{\mathrm{g}}}}$ and $\underline{R_\alpha}$ are rotation matrices:

$$\underline{R_\theta} = \begin{pmatrix} 1 & 0 & 0 \\ 0 & \cos\theta & \sin\theta \\ 0 & -\sin\theta & \cos\theta \end{pmatrix} ,$$

$$\underline{R_\varphi} = \begin{pmatrix} \cos\varphi & \sin\varphi & 0 \\ -\sin\varphi & \cos\varphi & 0 \\ 0 & 0 & 1 \end{pmatrix} ,$$

$$\underline{R_{\varphi_{\mathrm{g}}}} = \begin{pmatrix} \cos\varphi_{\mathrm{g}} & \sin\varphi_{\mathrm{g}} & 0 \\ -\sin\varphi_{\mathrm{g}} & \cos\varphi_{\mathrm{g}} & 0 \\ 0 & 0 & 1 \end{pmatrix}$$

and

$$\underline{R_\alpha} = \begin{pmatrix} \cos\alpha & 0 & \sin\alpha \\ 0 & 1 & 0 \\ -\sin\alpha & 0 & \cos\alpha \end{pmatrix} .$$

Once we have the tensor form factor, the scattering amplitude tensor ($\underline{A}$) is obtained by the summation of the form factor over several (theoretically infinite) number of layers. The tensor elements reduce to delta functions (these define the magnitudes of the scattering vectors)

multiplied by some factor, the norm of which is related to the scattering intensity at a given polarization of the scattered waves in dependence of the incident polarization of waves.

The intensity of the scattered light is calculated for the polarization of the incident and scattered light being either in the scattering plane (π-polarization) or perpendicular to it (σ-polarization). There are thus four possibilities to check for each RSoXS peak. The unit vectors defining the direction of the σ and π polarization can be deduced from Supplementary Figure 6b:

$$\vec{\pi}_{\mathrm{in}} = \left(\sin\left(\frac{\theta_{\mathrm{sc}}}{2}\right), 0, \cos\left(\frac{\theta_{\mathrm{sc}}}{2}\right)\right) \quad ,$$

$$\vec{\pi}_{\mathrm{sc}} = \left(-\sin\left(\frac{\theta_{\mathrm{sc}}}{2}\right), 0, \cos\left(\frac{\theta_{\mathrm{sc}}}{2}\right)\right) \quad ,$$

$$\vec{\sigma}_{\mathrm{in}} = (0,1,0) \quad ,$$

$$\vec{\sigma}_{\mathrm{sc}} = (0,1,0) \quad .$$

The scattering angle (θ_{sc}) is related to the scattering vector magnitude (q):

$$\sin\left(\frac{\theta_{\mathrm{sc}}}{2}\right) = \frac{q}{2k_0} \quad ,$$

where k_0 is the magnitude of the wave vector of the incident/scattered light. The intensities of the peaks I_{nm}, where $n = \sigma, \pi$ denotes the polarization of the scattered light and $m = \sigma, \pi$ polarization of the incident light, are (*1*):

$$I_{\sigma\sigma} = \left|\vec{\sigma}_{\mathrm{sc}} \cdot \underline{A} \cdot \vec{\sigma}_{\mathrm{in}}\right|^2 \quad ,$$

$$I_{\pi\sigma} = \left|\vec{\pi}_{\mathrm{sc}} \cdot \underline{A} \cdot \vec{\sigma}_{\mathrm{in}}\right|^2 \quad ,$$

$$I_{\sigma\pi} = \left|\vec{\sigma}_{\mathrm{sc}} \cdot \underline{A} \cdot \vec{\pi}_{\mathrm{in}}\right|^2 \quad ,$$

$$I_{\pi\pi} = \left|\vec{\pi}_{\mathrm{sc}} \cdot \underline{A} \cdot \vec{\pi}_{\mathrm{in}}\right|^2 \quad .$$

Below we study the scattering intensities and polarization of the scattered peaks for the four-layer and bilayer modulated smectic structures.

Four – layer structure

The tensor form factor of the ferri-like 4-layer structure is obtained by summing up the form factors (eq. (1)) by taking into the account the orientation of the long molecular axis in each smectic layer (Supplementary Figure 7) and by considering the phase difference due to the scattering at different layers, the thickness of each being d_0. Within each layer we put two resonant scatterers: one in the center of the lower and one in the center of the upper arm of the molecule. We assume, that, in general, they can have slightly different polarizability (different f_0) and that the neighboring arms of molecules from two different layers have the same polarizability. The form factors for these two types of dipoles are denoted by $\underline{F_1}$ and $\underline{F_2}$ (i.e., $f_0 = f_{01}$ for one dipole and $f_0 = f_{02}$ for the other dipole). The tilt angle θ and the apex angle

$\pi - 2\alpha$ are the same for all layers. The tilt of the molecular arm (α) is opposite in the lower and upper arm of the molecule. First, we assume that there is no general tilt, which means that the rotation matrix $\underline{R}_{\varphi_g}$ is a unit matrix.

The scattering amplitude tensor ($\underline{A}_4$) is obtained as a combination of the form factor due to a four layer structure and structure factor due to the 4-layer repeating unit:

$$\begin{aligned}\underline{A}_4 = \sum_{j=1}^{N} \big(& F_1(\theta, \alpha, 4j\varepsilon) + F_2(\theta, -\alpha, 4j\varepsilon)e^{iqd_0/2} + F_2(\theta, \alpha, \delta + (4j+1)\varepsilon)e^{iqd_0} \\ & + F_1(\theta, -\alpha, \delta + (4j+1)\varepsilon)e^{3iqd_0/2} \\ & + F_1(\theta, \alpha, \pi + (4j+2)\varepsilon)e^{i2qd_0} \\ & + F_2(\theta, -\alpha, \pi + (4j+2)\varepsilon)e^{i5qd_0/2} \\ & + F_2(\theta, \alpha, \pi + \delta + (4j+3)\varepsilon)e^{i3qd_0} \\ & + F_1(\theta, -\alpha, \pi + \delta + (4j+3)\varepsilon)e^{i7qd_0/2}\big)e^{4ijqd_0} \quad ,\end{aligned} \tag{2}$$

where N is the number of layers on which x-rays are scattered. The magnitude of the scattering vector is chosen as

$$q = \frac{2\pi}{d_0} h \quad , \tag{3}$$

where h is the Miller index. Because the basic periodicity is $4d_0$ (if $\varepsilon = 0$), one expects interference peaks at multiples of $1/4$. When $\varepsilon \neq 0$, these peaks split.

In eq. (2), the summation over a very large (infinite) number of layers leads to delta functions and the tensor elements are different from zero only for $\pm 8\varepsilon + 8\pi h = 2\pi m$ and $\pm 4\varepsilon + 8\pi h = 2\pi m$, where m is an integer, which means:

$$h = \frac{m}{4} \pm \frac{\varepsilon}{\pi}$$

and

$$h = \frac{m}{4} \pm \frac{\varepsilon}{2\pi} \quad .$$

We are interested in the integer m being $0, 1$ or 2. The magnitudes of the scattering vectors related to the allowed peaks are denoted by $q_2 \pm q_\mathrm{m}$, $q_2 \pm 2q_\mathrm{m}$, $q_4 \pm q_\mathrm{m}$, $q_4 \pm 2q_\mathrm{m}$, q_m and $2q_\mathrm{m}$, where $q_4 = \pi/(2d_0)$, $q_2 = \pi/d_0$ and $q_\mathrm{m} = \varepsilon/d_0$.

Let us write the scattering amplitude tensor $\underline{A}_4$ in a general form:

$$\underline{A}_4 = \begin{pmatrix} f_{11} & f_{12} & f_{13} \\ f_{21} & f_{22} & f_{23} \\ f_{31} & f_{32} & f_{33} \end{pmatrix} \quad .$$

The summation over j in eq.(2) gives that the tensor elements f_{11}, f_{22}, f_{12} and f_{21} are different from zero at $h = \frac{m}{4} \pm \frac{\varepsilon}{\pi}$, f_{11} and f_{22} also at $h = \frac{m}{4}$, f_{33} only at $h = \frac{m}{4}$, while f_{13}, f_{31}, f_{23} and f_{32} are different from zero only at $h = \frac{m}{4} \pm \frac{\varepsilon}{2\pi}$.

We present the results for the intensities of the peaks assuming symmetric molecules ($f_{01} = f_{02}$). The ratio of the intensities (I_{4-}/I_{4+}) of the peaks at $q_4 \pm q_\mathrm{m}$ as a function of angle δ is given in Supplementary Figure 8a. For the presentation of the results we have chosen $\theta = 0.25$ (the experimentally estimated value) and $\varepsilon = 0.5$ (the value in the middle of the temperature range of the modulated smectic phase). Experimentally, the intensity of the $q_4 + q_\mathrm{m}$ peak is by an order of magnitude higher than the intensity of the $q_4 - q_\mathrm{m}$ peak. We have added a line to the plot at $I_{4-}/I_{4+} = 0.05$, which corresponds to the intensity of the $q_4 + q_\mathrm{m}$ peak being 20-times higher than the intensity of the $q_4 - q_\mathrm{m}$ peak. We see, that the value of δ at which the intensity ratio crosses this line depends on the value of α and it is different for structures with opposite layer chirality. We see, that we obtain the proper ratio of the $q_4 \pm q_\mathrm{m}$ intensities if the angle δ is larger than 1. This value seems reasonable, because the modulated smectic structure is formed below the twist-bend nematic phase in which one observes a strong resonant peak due to ideal helix with pitch length approaching the four – layer periodicity. We note, that the value of δ at which the intensity ratio curve crosses the $I_{4-}/I_{4+} = 0.05$ line slightly depends also on the value of ε. In presentation of further results we use $\delta = 1.2$.

Supplementary Figures 8b,c give the intensities of the peaks $2q_\mathrm{m}$, $q_4 \pm q_\mathrm{m}$ and $q_2 \pm q_\mathrm{m}$. The peaks q_m, $q_4 \pm 2q_\mathrm{m}$ and $q_2 \pm q_\mathrm{m}$ are zero in this model, also if asymmetry is considered. If a general tilt is considered, as well, then the intensity of these peaks becomes different from zero. One special case of the general tilt is $\varphi_\mathrm{g} = \pi$ in every second layer. This means that there is a layer chirality switch from one layer to another. Because there are no experimentally observed q_m, $q_4 \pm 2q_\mathrm{m}$ and $q_2 \pm q_\mathrm{m}$ peaks, we conclude, that the general tilt, if present, is very small and that there is no chirality switch from one layer to another. From Supplementary Figures 8b,c we see, that the peak intensities vary with α and θ. Because no splitting of the q_2 peak is observed experimentally, we expect the intensities of the $q_2 \pm 2q_\mathrm{m}$ peaks to be much lower than the intensities of the $q_4 \pm q_\mathrm{m}$ peaks. They are lower if α is small, so we conclude, that the apex angle of molecules in material *D1* that forms a 4-layer structure is close to π. In addition, we can expect that α is positive in order to have the $q_4 - q_\mathrm{m}$ stronger than the $q_2 \pm 2q_\mathrm{m}$ peaks (see Supplementary Figure 8b). The intensities of the $q_2 \pm 2q_\mathrm{m}$ peaks reduce also by reducing the angle δ. The change in the modulation angle ε does not have a significant effect on the intensities (and the ratio between them).

Figure 3f in the main text shows the orientation of molecules as a function of ε in a stack of several layers in order to visualize the temperature development of the helical structure. We see, that the system tries to unwind into a simple anticlinic structure, although this situation is not experimentally reachable. A video clip showing the development of the structure by a continuous increase of ε is available as Supplementary Movie 1.

Bilayer structure

To model the bilayer structure, we model the director positions on the cone as shown in Supplementary Figure 7b. The scattering amplitude tensor ($\underline{A}_2$) is obtained by following the procedure given in the construction of the 4-layer structure (see the text above eq. (2)).

In eq. (4), the summation over a very large (infinite) number of layers leads to delta functions and the tensor elements are different from zero only for $\pm 4\varepsilon + 4\pi h = 2\pi m$ and $\pm 2\varepsilon + 4\pi h = 2\pi m$, where m is an integer, which means:

$$h = \frac{m}{2} \pm \frac{\varepsilon}{\pi}$$

and

$$h = \frac{m}{2} \pm \frac{\varepsilon}{2\pi} \; ,$$

where we are interested in the integer m being 0 or 1. The magnitudes of the scattering vectors related to the allowed peaks are denoted by $q_2 \pm q_{\mathrm{m}}$, $q_2 \pm 2q_{\mathrm{m}}$, q_{m} and $2q_{\mathrm{m}}$, where $q_2 = \pi/d_0$ and $q_{\mathrm{m}} = \varepsilon/d_0$.

Supplementary Figure 9 shows the intensities of the $q_2 \pm q_{\mathrm{m}}$, $q_2 \pm 2q_{\mathrm{m}}$, q_{m} and $2q_{\mathrm{m}}$ peaks at experimental values of θ and ε ($\theta = 0.4$, $\varepsilon = 0.3$) as a function of δ. For α we take $\alpha = \pm\pi/6$ (the apex angle is 120 deg). Because experimentally no $q_2 \pm 2q_{\mathrm{m}}$ and q_{m} peaks are observed, from graphs in Supplementary Figure 8 we conclude that $\delta \approx 0$, i.e. the structure is anticlinic with the helical modulation superimposed over it. We also see that the layer chirality determines the wave vector of the peak with the highest intensity. If $\alpha < 0$ (this is equivalent to positive α and $\varphi_{\mathrm{g}} = \pi$) this is the peak with $q = q_2 + q_{\mathrm{m}}$, while if $\alpha > 0$, the peak with $q = q_2 - q_{\mathrm{m}}$ has the highest intensity. In experiment, the peak with $q = q_2 + q_{\mathrm{m}}$ has higher intensity than the peak with $q = q_2 - q_{\mathrm{m}}$, so we conclude that $\alpha < 0$.

Supplementary References

1. D. H. Templeton, L. K. Templeton, *Acta Crystallogr. Sect. A* **36**, 237-241 (1980).
2. V. E. Dmitrienko, *Acta Crystallogr. Sect. A* **39**, 29-35 (1983).
3. A. M. Levelut, B. Pansu, *Phys. Rev. E* **60**, 6803-6815 (1999).
