# Supplementary material for: Multi-level chirality in liquid crystals formed by achiral molecules
Source: Nat Commun. 2019 Apr 23;10:1922. doi: 10.1038/s41467-019-09862-y (PMC6478950; doi:10.1038/s41467-019-09862-y)
Supplement: Supplementary file 3 — Description of Additional Supplementary Files [file 41467_2019_9862_MOESM3_ESM.pdf]

Description of Additional Supplementary Files

File Name: Supplementary Movie 1
Description: Gradual evolution of the 4-layer structure of the smectic phase due to increasing angle ε at constant angles δ = 1.2 and θ = π/3. The cone angle θ is enlarged with respect to its experimentally determined value in order to better visualize the rotation of the molecules.
