# Supplementary figures and images for: Multi-level chirality in liquid crystals formed by achiral molecules
Source: Nat Commun. 2019 Apr 23;10:1922. doi: 10.1038/s41467-019-09862-y (PMC6478950; doi:10.1038/s41467-019-09862-y)

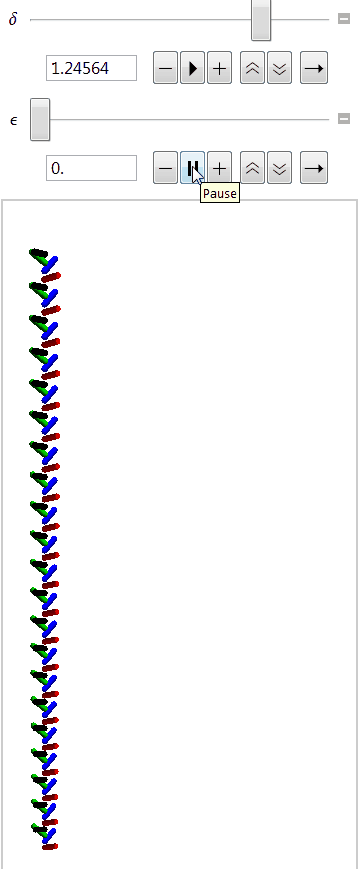

Supplement: Supplementary file 4 — Supplementary Movie 1 [file 41467_2019_9862_MOESM4_ESM.gif]
